# Supplementary material for: Arctigenin Attenuates Vascular Inflammation Induced by High Salt through TMEM16A/ESM1/VCAM-1 Pathway
Source: Biomedicines. 2022 Oct 31;10(11):2760. doi: 10.3390/biomedicines10112760 (PMC9687712; doi:10.3390/biomedicines10112760)
Supplement: Supplementary file 1 [file biomedicines-10-02760-s001.zip › biomedicines-1913494-supplementary.pdf]

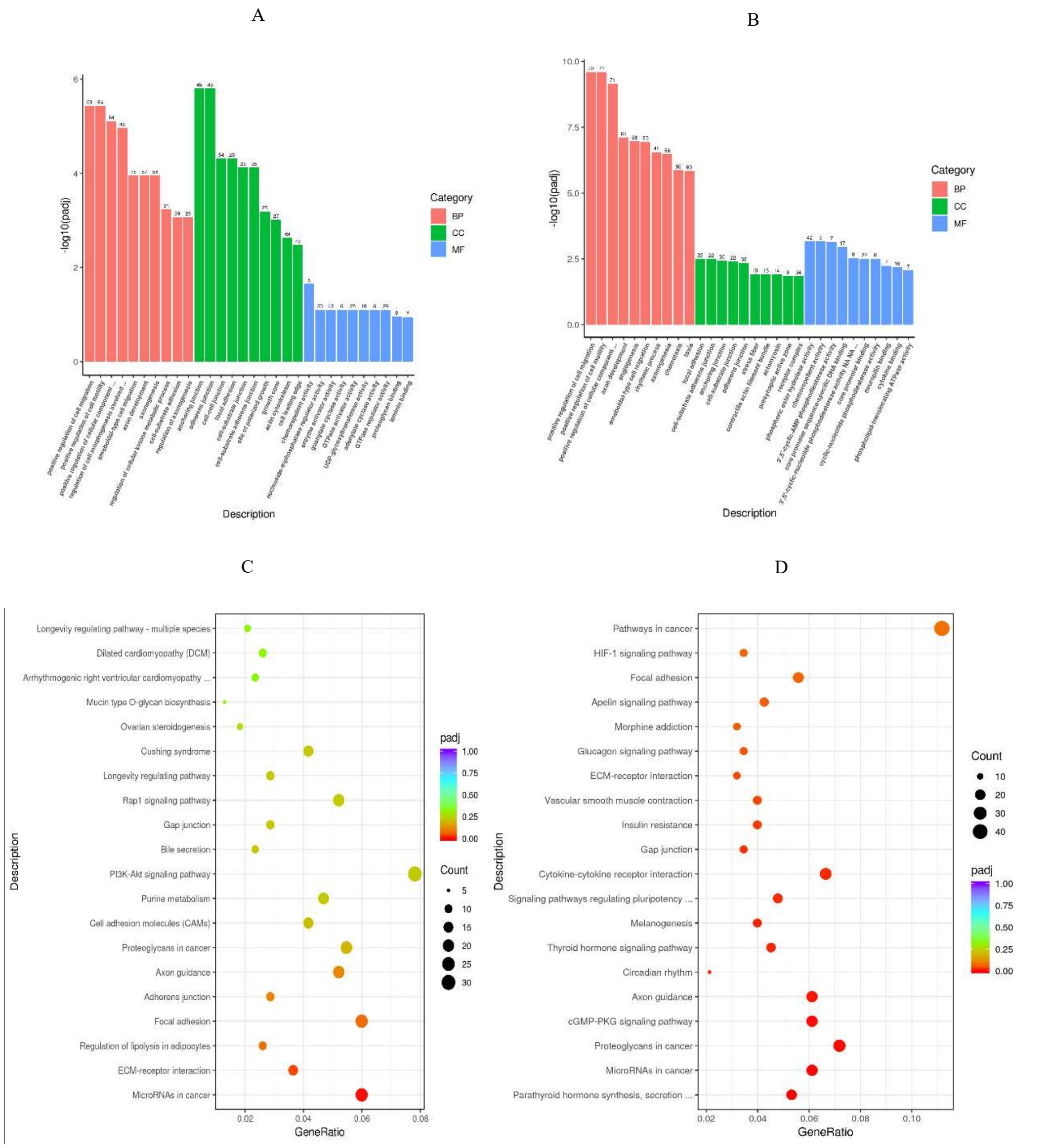

Supplementary Figure S1: A&B: Bar chats of Gene Ontology (GO) enrichment analysis of differentially expressed downstream genes of TMEM16A, the abscissa represented the GO terms, and the ordinate represented the number of differentially genes (A: HS & ARC, B: HS & T16). C&D: Dot plots of Kyoto Encyclopedia of Genes and Genomes (KEGG) enrichment analysis of differentially expressed downstream genes of TMEM16A, the abscissa represented the ratio of number of differentially genes in KEGG pathway to total differentially genes, and the ordinate represented pathways. The color gradient from red to purple indicated P-value changed from small to large after correction. (C: HS & ARC, D: HS & T16). NS: normal salt group, HS: high salt group, ARC: HS+ arctigenin (100mg/kg) group, T16: HS+ T16Ainh-A01 group.

A

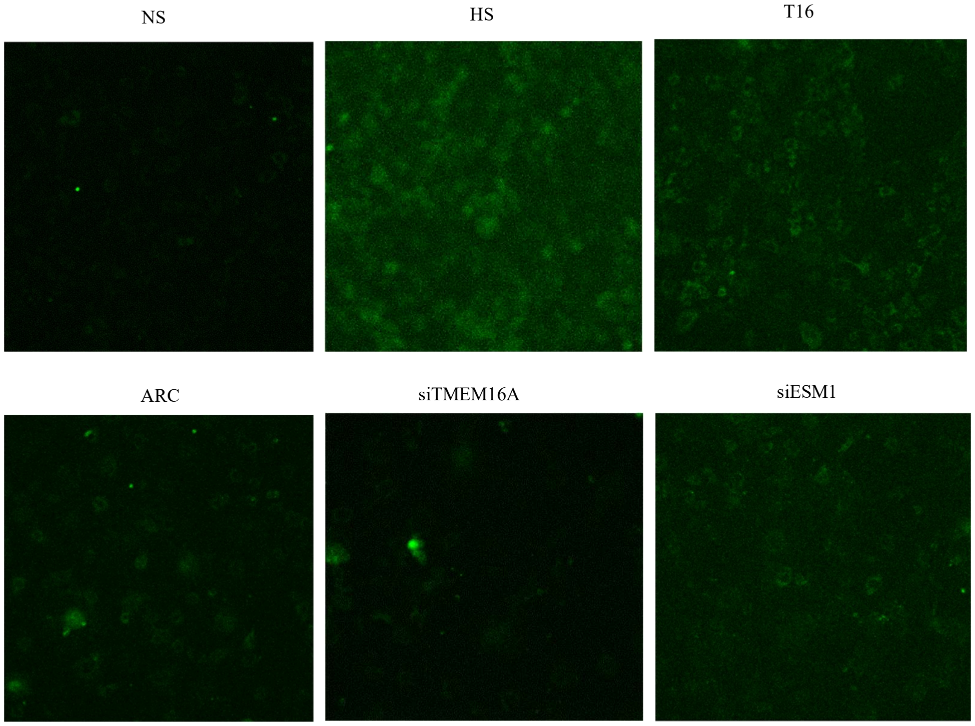

B

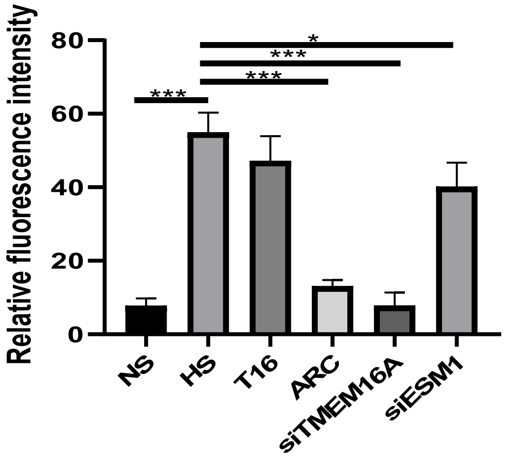

Supplementary Figure S2A: Adhesion assay of Calcein-AM-labled macrophages (green) adherent for VSMCs with Immunofluorescence stanining in different groups. **Supplementary Figure S2B**: Relative fluorescence were quantified. ***P<0.05, *P <0.05; t-test (n=3). NS: normal salt group, HS: high salt group, T16: HS+ T16Ainh-A01 group, ARC: HS+ arctigenin (100mg/kg) group, siTMEM16A: HS+siRNA TMEM16A group. siEMS1: HS+ siRNA ESM1 group.
